# Supplementary material for: Dual-layer optical encryption fluorescent polymer waveguide chip based on optical pulse-code modulation technique
Source: Nat Commun. 2023 Jul 29;14:4578. doi: 10.1038/s41467-023-40341-7 (PMC10387099; doi:10.1038/s41467-023-40341-7)
Supplement: Supplementary file 1 — Supplementary Information [file 41467_2023_40341_MOESM1_ESM.pdf]

Supplementary Information

Dual-layer optical encryption fluorescent polymer waveguide chip based on optical pulse-code modulation technique

Chunxue Wang[1], Daming Zhang[1], Jian Yue[1], Xucheng Zhang[1], Hang Lin[1], Xiangyi Sun[1], Anqi Cui[1], Tong Zhang[1], Changming Chen[1]*, and Teng Fei[1]*

[1]State Key Laboratory of Integrated Optoelectronics, College of Electronic Science and Engineering, Jilin University, Changchun, 130012, PR China
E-mail: chencm@jlu.edu.cn and feiteng@jlu.edu.cn

Supplementary Information

Supplementary Figure S1. The optical waveguide coupling test system.

Optical pulse-code modulation coupling testing system

An optical pulse-code modulation coupling testing system was built as illustrated in **Figure S1**, then the optical characteristics and dynamic modulation response characteristics of the dual-layer encryption waveguide device were measured.

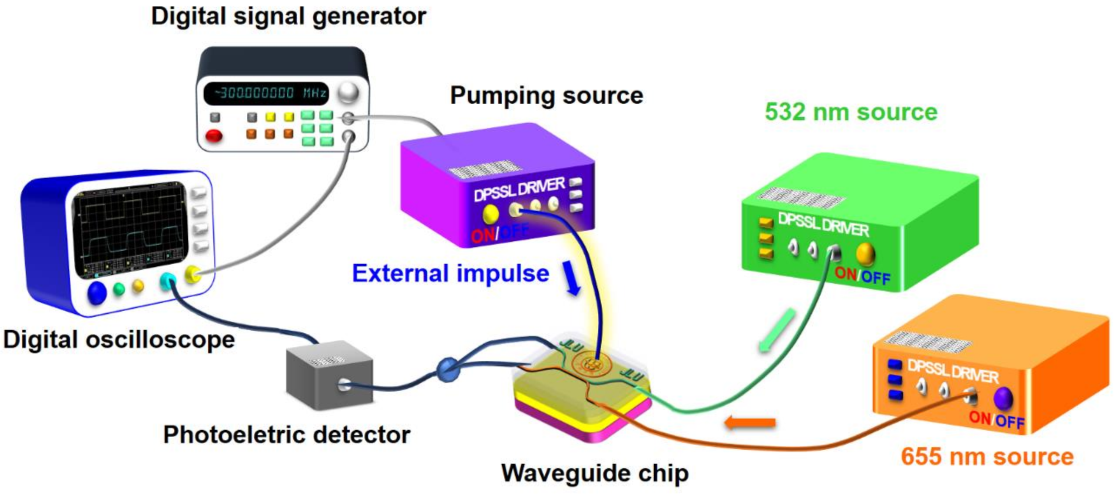

Figure S1. The optical waveguide coupling test system of the dual-layer encryption waveguide chip.
